# Neuropeptide substance P alters stem cell fate to aid wound healing and promote epidermal stratification through asymmetric stem cell divisions

**DOI:** 10.1093/stmcls/sxae009

**Published:** 2024-02-01

**Authors:** Ayman Khalifa, Tong Xiao, Brook Abegaze, Tracy Weisenberger, Alexandra Charruyer, Samia Sanad, Taher AbuElnasr, Sakeen W Kashem, Marlys Fassett, Ruby Ghadially

**Affiliations:** Zoology Department, Faculty of Science, Zagazig University, Egypt; Department of Dermatology, UCSF, San Francisco, CA, USA; VA Medical Center, San Francisco, CA, USA; Department of Dermatology, UCSF, San Francisco, CA, USA; VA Medical Center, San Francisco, CA, USA; Department of Dermatology, The Second Affiliated Hospital of Xi’an Jiaotong University, Shaanxi, People’s Republic of China; Department of Dermatology, UCSF, San Francisco, CA, USA; VA Medical Center, San Francisco, CA, USA; Department of Dermatology, UCSF, San Francisco, CA, USA; VA Medical Center, San Francisco, CA, USA; Department of Dermatology, UCSF, San Francisco, CA, USA; VA Medical Center, San Francisco, CA, USA; Zoology Department, Faculty of Science, Zagazig University, Egypt; Zoology Department, Faculty of Science, Zagazig University, Egypt; Department of Dermatology, UCSF, San Francisco, CA, USA; Department of Dermatology, UCSF, San Francisco, CA, USA; Department of Dermatology, UCSF, San Francisco, CA, USA; VA Medical Center, San Francisco, CA, USA

**Keywords:** adult stem cells, epidermis, progenitor cells, proliferation, self-renewal, stem cells

## Abstract

Loss of sensory innervation delays wound healing and administration of the neuropeptide substance P improves re-epithelialization. Keratinocyte hyperproliferation postwounding may result from symmetric stem cell (SC) self-renewal, asymmetric SC self-renewal, committed progenitor divisions, or a combination of these. However, the effects of sensory denervation and of neuropeptides on SC proliferation are not known. Here we show that early after wounding both asymmetric and symmetric SC self-renewal increase, without significant committed progenitor (CP) activation. Decreased sensory innervation is associated with a decrease in both SC and CP proliferation. Based on previous work showing that substance P is decreased in capsaicin-treated mice and improves wound healing in normal skin, we examined the effects of substance P on SC and CP proliferation during wound healing. Substance P restored asymmetric SC proliferation in skin with decreased sensory innervation, both at baseline and following wounding. Epidermis with decreased sensory innervation was severely thinned. Consistent with this, substance P-induced asymmetric SC proliferation resulted in increased stratification in skin with both normal and decreased innervation. Lapatinib prevented the substance P-induced increase in asymmetric SC divisions in murine epidermis, as well as the increase in epidermal stratification, suggesting that asymmetric SC divisions are required for epidermal stratification.

## Graphical Abstract

**Graphical Abstract:**
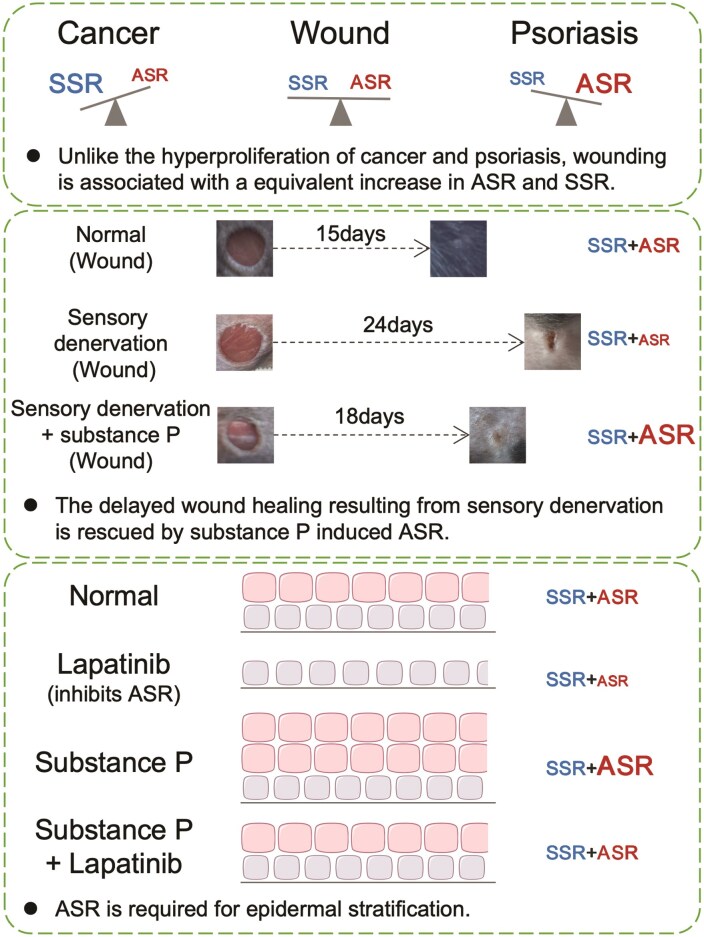

**Significance Statement**

Symmetric stem cell self-renewal underlies aggressive cancers and asymmetric self-renewal underlies hyperproliferation in psoriasis. We found an equivalent increase in asymmetric and symmetric SC self-renewal in wound healing. In skin with decreased sensory innervation, there was decreased stem and progenitor proliferation and substance P improves wound healing through increased asymmetric divisions. These studies are key to the goal of manipulating stem cell divisions for therapy of wounds in skin with decreased sensory innervation, as in diabetes or aging. These studies also provide support for the concept that stratification requires asymmetric stem cell division rather than the converse.

## Introduction

Loss of sensory innervation in mice and rats is associated with impaired wound healing^1-3^ and neuropeptides can improve wound healing.^4-7^

Re-epithelialization after wounding requires SC self-renewal and differentiation. In the basal layer, SC self-renewal involves asymmetric and symmetric divisions. Asymmetric divisions promote stratification through production of one suprabasal non-SC committed progenitor (CP, aka transit amplifying cell), as well as a replacement SC in the basal layer.^8-12^ Symmetric SC divisions lead either to 2 SCs (symmetric SC self-renewal) or 2 differentiated CPs (symmetric SC differentiation). CP divisions are believed to lead to terminal differentiation after a limited number of divisions.^13^ After wounding of murine tail, results of the analysis of labeled keratinocyte clones suggested that SCs undergo predominantly asymmetric self-renewal divisions.^14^

Alterations in SC behavior that occur with loss of sensory innervation and the associated decrease in neuropeptide levels are not well understood. Loss of sensory innervation (capsaicin-induced) impairs the egress of SC progeny from hair follicles after wounding.^15^ In denervated wounds, Lgr6-positive SCs proliferate less actively and have a higher propensity to terminally differentiate.^16^ Denervation results in a reduction of the ability of bulge cells to become epidermal SCs after wounding.^17^ Surgical denervation of the touch dome in mice resulted in the loss of Gli1 + touch dome SCs.^18^ Denervation impacts SC behavior in multiple systems. Denervation of the parasympathetic submandibular ganglion (SMG) impaired SMG morphogenesis and proliferation of progenitor cells in mice.^19^ Denervation of nonmyelinated Schwann cells in the bone marrow of adult mice resulted in the loss of hematopoietic SCs.^20^ These findings indicate that innervation is important in the modulation of SC physiology during wound healing.

Substance P is a neuropeptide agonist of the neurokinin-1 receptor, a G-protein-coupled-receptor.^21^ Substance P secretion is directly associated with sensory innervation. Decreased sensory innervation results in depletion of substance P^22-24^ and increased cutaneous nerves in KC-Tie2 mice were associated with a 4-fold increase in substance P.^25^ This association suggests new therapeutic strategies for hyperproliferative disorders such as psoriasis.^25^

Substance P improves wound healing in skin.^5,26-29^ Substance P appears to impact all phases of wound healing and has been found to intensify inflammatory responses, promote angiogenesis, and increase proliferation and migration of keratinocytes and fibroblasts.^21^ The application of substance P results in improved wound healing in multiple models.^5,26-29^ Substance P significantly increases the proliferation and migration of cells and decreases wound area after 24 h in vivo.^30^ Furthermore, an antagonist of the neurokinin-1 receptor, paired with substance P, inhibited the improved wound healing, diminishing the migration of cells and increasing wound area significantly in comparison to the control group.^30^

Epidermal pathology in both human and murine models of psoriasis and cancer, and in human aging, is associated with alterations in SC fate, and rebalancing SC fate ameliorated the disease phenotype.^8,31^ Here we studied SC division fate and proliferation after wounding, in both normal epidermis and epidermis with decreased sensory innervation. We then examined effects of neuropeptide substance P. Understanding changes in SC and CP behavior with wounding in normal skin and skin with decreased sensory innervation could provide strategies for rebalancing SC fate and improving re-epithelialization of wounds, especially in diabetes and aging. Unexpectedly, these studies also provided insight into the role of SC division fate in stratification.

## Methods

### Animals

Animal experiments (C57BL/6J mice) were approved by the IACUC of the San Francisco VAMC.

### Wounds

Mice (8-12 weeks), under isoflurane anesthesia, were shaved and 6 mm biopsy punches used to generate full-thickness wounds on each side of the dorsal trunk. Mice did not receive analgesics after initial wounding.^32^ Wounds were imaged and wound area was calculated using the ImageJ measurement tool.^33^

### Basal Keratinocyte Divisions in Tissue Sections

Formalin-fixed paraffin-embedded tissue sections (5-8 µm) were incubated with antibodies to α/γ tubulin (microtubules/centrioles) (Invitrogen 13-8000, Abcam Ab11316) at a dilution of 1:100 and 1:200, respectively. Alexa Fluor 488 secondary antibodies (InvitrogenA-11001) at a dilution of 1:500 to identify the spindle apparatus. The angle between spindle axis and basal membrane (angle of division) was measured using the angle tool in ImageJ. Divisions <30° were designated parallel and divisions with angles 60°-90° designated perpendicular.^10^ Spindle axes perpendicular to the basal membrane were considered asymmetric SC divisions.^10^ Basal layer length was measured using ImageJ measurement tool.

### Keratinocyte Stem Cell Division Fate In Vitro

Samples were obtained from skin in a 4-mm radius around the wound and from non-wounded (control) littermates. Keratinocytes isolated from freshly obtained C57BL/6J skin, using Dispase 25 U/mL, followed by 0.05% trypsin-EDTA, were plated (200,000 cells/0.7 cm^2^ chamber slide) and incubated in CnT-PR (Cellntec). Forty-eight hours later, cells were fixed with paraformaldehyde, then incubated with anti-Numb (goat, 1:100) and anti-cytokeratin 1 (rabbit, 1:200) antibodies (AbcamAb4147 and Ab9286), followed by Alexa Fluor 488 and 568 secondaries (Invitrogen, A-11001, A-11004) 1:500. Divisions were identified as post-mitotic sister pairs (DM4B fluorescence microscope, Leica). To confirm that sister pairs were post-mitotic, we determined the frequency of non-mitotic pairs. At the plated density, non-divided cells sitting together by chance (identified as red/green side by side non-sisters) was 5.1% of identified sister cells (1/58 or 1.7% red/green identified and a similar frequency of red/red and green/green side-by-side non-divided cells projected). Whenever possible, >100 divisions were analyzed per sample.

### Sensory Denervation

Anesthetized 2-week-old mice were injected with 0.1 mg/kg buprenorphine HCl and then capsaicin (Sigma-M2028) subcutaneously.^34-36^ Resiniferatoxin (RTX) was unavailable commercially. Two injections of capsaicin were administered, 21 (75 µg/kg) and 14 (50 µg/kg) days before wounding.^35^ Control mice received vehicle (Tween:ethanol:saline1:1:8/vol). Innervation was assessed using the tail-flick test (Supplementary Table S1).

### Treatment With Substance P

Media was supplemented with substance P (Sigma-Aldrich-S6883) 0-10^−3^ M (vehicle, 10^−9^ M, 10^−7^ M, 10^−5^ M, or 10^−3^ M),^5,37,38^ for 48 h. In vivo, mice were injected intradermally with 32 µg substance P in 200 µL PBS. Control mice received PBS. To study substance P and wound healing in skin with decreased sensory innervation, capsaicin-treated mice were treated with substance P or vehicle, injected intradermally around the wound every other day, for 10 days before wounding. One cohort of mice received substance P every other day until healed. The other cohort of mice received one further intradermal substance P treatment on day 2 after wounding, and then skin was harvested on day 4 and placed in culture with 200 ng/mL substance P or vehicle and then analyzed at 48 hours. For epidermal thickness, skin was fixed in 10% formaldehyde, paraffin embedded, and sectioned. Tissues were stained with H&E, photographed, and epidermal thickness measured using ImageJ. To minimize bias, 3 equidistant sites of 8 photographic fields were used for measurements.

To determine the effect of substance P on mice with normal sensory innervation and mice with decreased sensory innervation, capsaicin and vehicle-treated mice underwent intradermal injections of substance P or vehicle (PBS) every other day for 24 days before tissue harvest of intact (non-wounded) skin, that was then analyzed.

### Lapatinib and Substance P Treatment

For in vitro studies of murine keratinocytes, media was supplemented with substance P 10^−7^ M, Lapatinib 2.5 µM, substance P with lapatinib, or vehicle (DMSO), for 48 hours. Cells were then studied for expression of Numb and keratin 1, as described above. In vivo, substance P (200 µL, 10^−7^ M, Sigma Aldrich-S6883), lapatinib (50 µL, 2.5 µM, Santa Cruz-202205A),^39^ substance P with lapatinib, or vehicle (DMSO), was injected intradermally into mice every other day for 2 weeks. Skin biopsies were then taken from each flank for assessment of epidermal thickness and basal cell density.

### Basal Cell Density

For each sample, the mean number of cells present in 6-15 100 µm lengths was determined.

### Statistical Analysis

**Figure 1.**
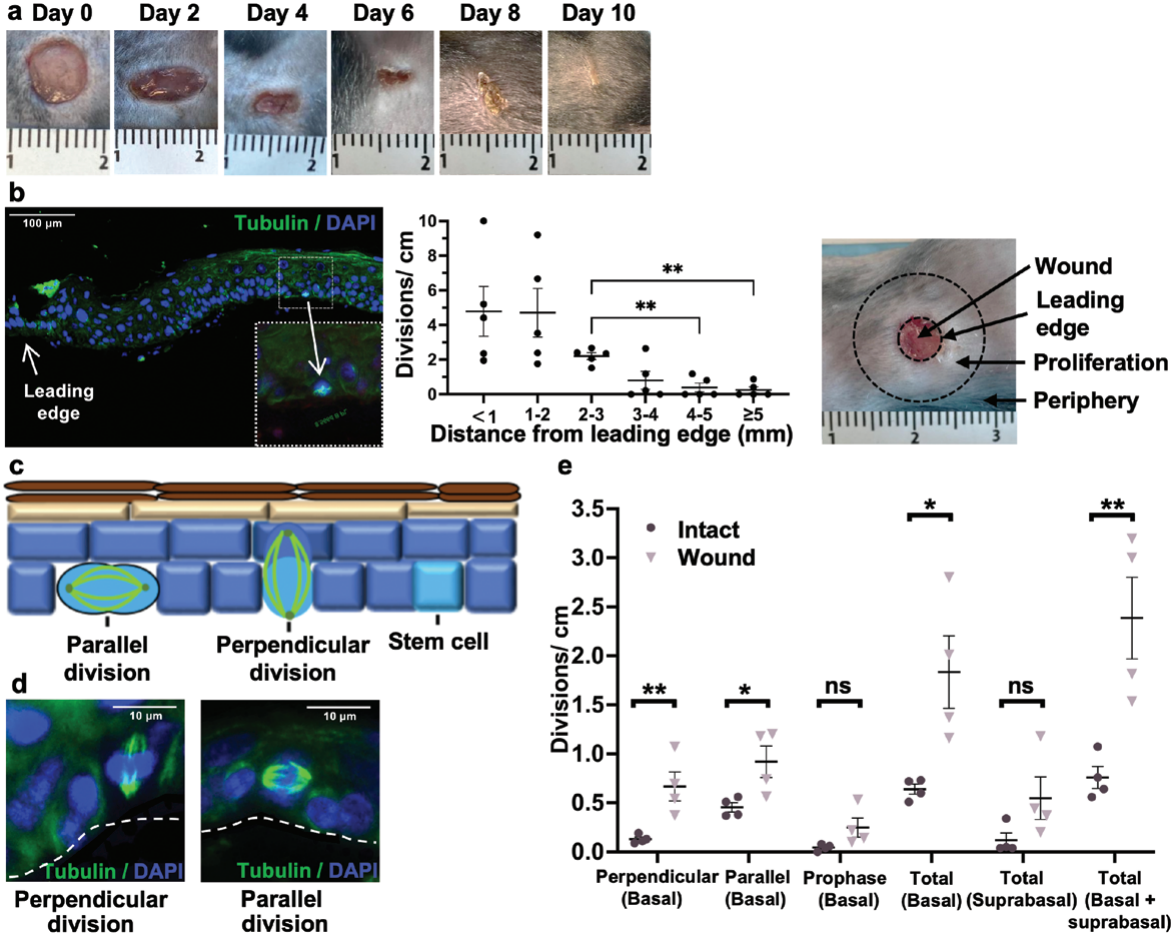
Perpendicular and parallel keratinocyte divisions in the basal layer, but not divisions in the suprabasal layer, are increased at day 4 after wound healing. (**a**) Photographic documentation of normal murine skin healing after wounding. Ruler in mm. (**b**) Immunofluorescence image of horizontal division (α/γ tubulin expression in microtubules/ centrioles) (left). Divisions peripheral to the wound (center) (*n* = 4). Wound, leading edge, and proliferation zone (right). (**c**) Schematic representation of murine epidermis, showing divisions both perpendicular and parallel to the basement membrane. (**d**) Immunofluorescence images of cell division angles in the basal layer. (**e**) Analysis of divisions in epidermis 4 days post-wounding vs. intact murine skin, in tissue sections (*n* = 4. Intact: 57 divisions. Wound: 130 divisions). Data are presented as mean ± SEM; *P < 0.05. **P < 0.01. ns; not significant.

**Figure 2.**
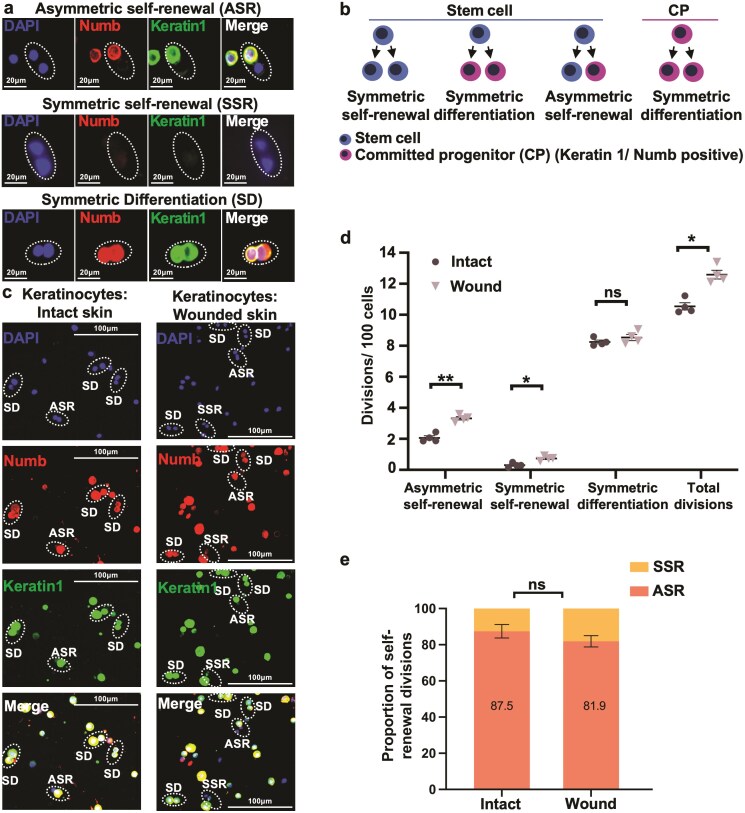
In normal mice, the hyperproliferation after wounding is associated with an increase in both asymmetric and symmetric SC self-renewal, with no detected change in CP divisions. (**a**) Division analysis in vitro using Numb and keratin 1 (present in the differentiated cell of asymmetric divisions), and DAPI. (**b**) Schematic of SC and CP division fates observed in keratinocytes. (**c**) Illustrative samples of cell divisions in keratinocytes from intact and wounded skin, in vitro. (**d**) SC divisions in vitro 4 days post wounding (*n* = 4). Skin was harvested in a 4-mm radius around the wound to include the proliferative zone. Control skin was harvested from littermates. Keratinocytes were isolated, plated (200,000 cells/0.7 cm^2^ chamber slide) in culture media, and Numb and keratin 1 expression studied using an immunofluorescence microscope. (**e**) The proportions of symmetric and asymmetric SC divisions were not found to be significantly altered in wounded skin vs. intact skin (*n* = 4). Data are presented as mean ± SEM; *P < 0.05. **P < 0.01. ns; not significant.

**Figure 3.**
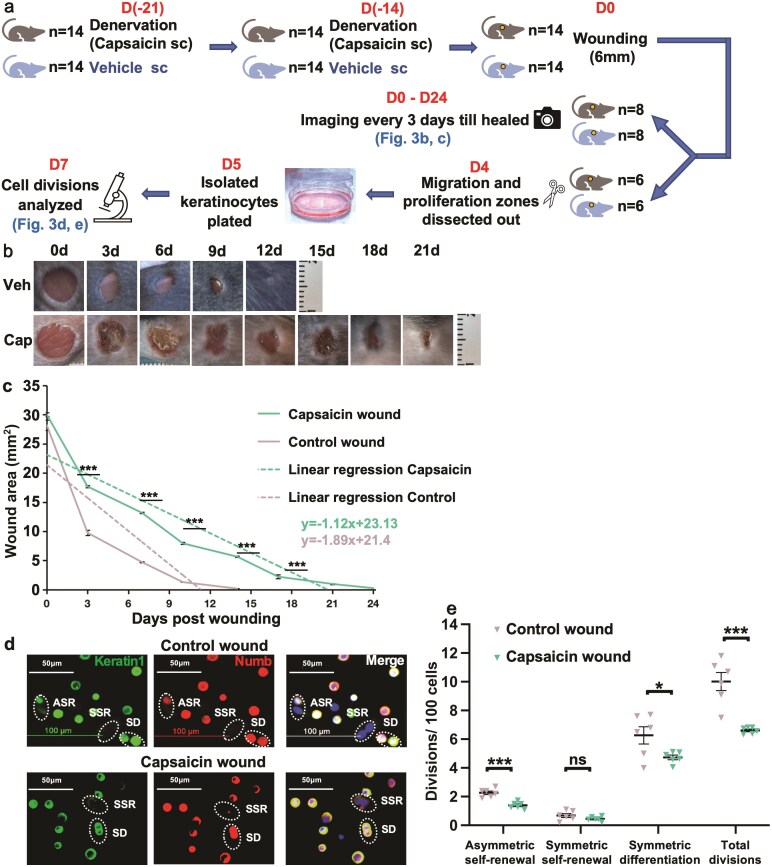
The decreased proliferative response to wounding in mice with decreased sensory innervation is associated with a decrease in asymmetric SC self-renewal and CP symmetric differentiation. (**a**) Experimental design. Neonatal mice were subjected to sensory denervation before wounding (day 0). Post-wounding, one cohort was followed to assess healing and one cohort was sacrificed at 4 days to assess cell division fates in vitro. (**b**) Representative photographic documentation of delayed wound healing in mice with decreased sensory innervation. (**c**) Wound area and rate of wound healing in mice with decreased sensory innervation vs. control mice. (**d**) Representative images of division fate analysis in mice with wounds from mice with decreased sensory innervation vs. controls. (**e**) SC and CP proliferative response to wounding with decreased sensory innervation vs. intact sensation. Data are presented as mean ± SEM; *P < 0.05. ***P < 0.001. ns; not significant. s.c.; subcutaneously. Con; control. Cap; capsaicin.

**Figure 4.**
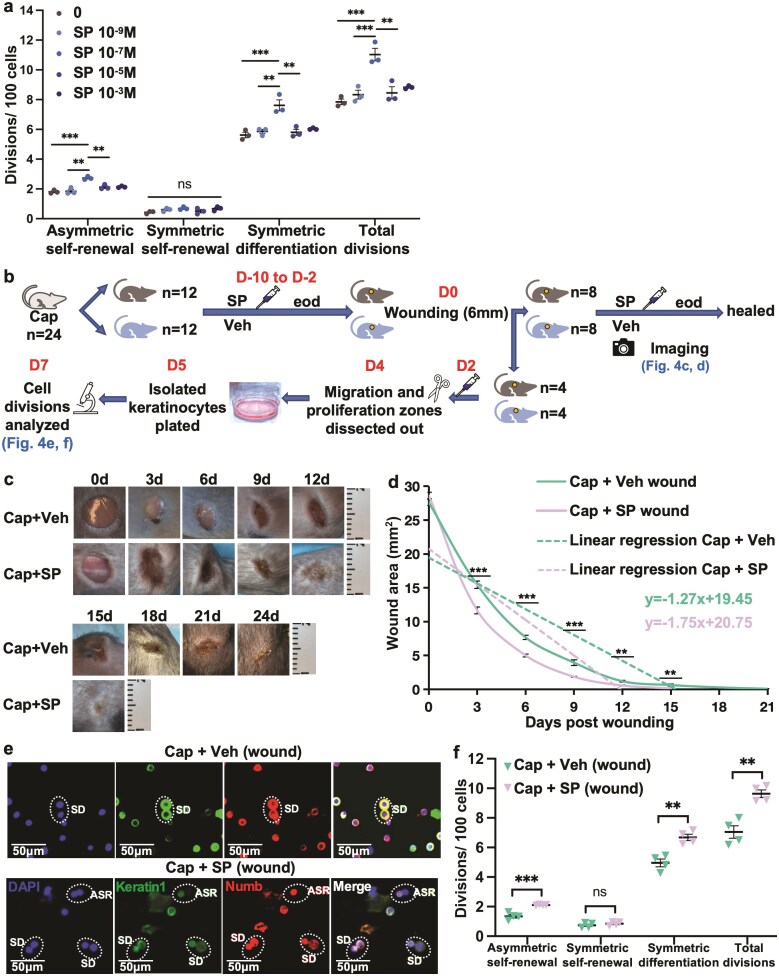
Substance P improves wound healing in mice with decreased sensory innervation and increases keratinocyte asymmetric SC divisions and CP symmetric differentiation divisions in vitro. (**a**) Dose-response assessment of the effect of substance P on cell divisions (*n* = 3). (**b**) Experimental design: Mice with decreased sensory innervation were given SP or vehicle intradermally every other day (eod) for 5 injections as well as at the time of wounding on day 0. One cohort then continued to receive SP or vehicle eod till the wound was healed. Another cohort received intradermal SP on day 2 post-wounding and skin was harvested on day 4 and placed in culture with 200 ng/mL SP or vehicle. At 48 hours cells were harvested for cell division analysis. (**c**) Representative photographic documentation of the improved healing seen with substance P treatment. (**d**) Effect of substance P on wound area and rate of wound healing in mice with decreased sensory innervation (*n* = 8). (**e**) Representative images of division fate analysis using Numb and keratin 1 immunofluorescence, in substance P vs. vehicle-treated wounds, in mice with decreased sensory innervation. Representative images. (**f**) SC and CP proliferative responses to substance P vs. vehicle treatment of wounds, in mice with decreased sensory innervation. Data are presented as mean ± SEM. **; *P* < 0.01. ***; *P *< 0.001. ns; not significant. Veh; vehicle. Cap; capsaicin. SP; substance P. eod; every other day.

**Figure 5.**
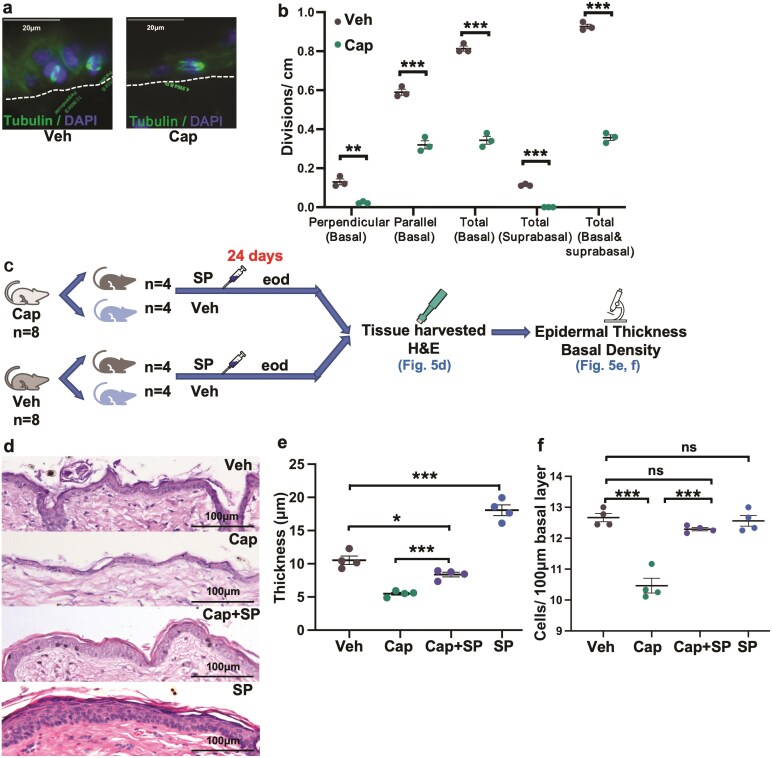
Substance P administration can ameliorate the decreases in perpendicular and parallel basal layer divisions, epidermal stratification, and basal cell density in skin with decreased sensory innervation. (**a**) Representative images (α/γ tubulin fluorescence) of tissue sections from non-wounded skin with decreased sensory innervation vs. intact sensory innervation. Perpendicular (asymmetric SC) division (left). Parallel division (right). (**b**) Division analysis in non-wounded epidermis with decreased sensory innervation vs. intact sensory innervation (*n* = 3). (**c**) Experimental design: Mice with decreased sensory innervation and vehicle-treated mice underwent intradermal injections of SP or vehicle eod for 24 days before tissue harvest of intact (non-wounded) skin, for study of epidermal thickness and basal cell density. (**d**) Representative histology of skin with decreased sensory innervation (Cap), decreased sensory innervation treated with substance P (Cap + SP), as well as capsaicin vehicle (Veh)-treated skin and skin treated with Cap vehicle and SP (*n* = 4). (**e**) Epidermal thickness in micrometer (samples as in c). (**f**) Basal cell density (cells/100 µm) (samples as in c). Data are presented as mean ± SEM. *P < 0.05. **P < 0.01. ***P < 0.001. Veh; vehicle. Cap; capsaicin. SP; substance P. eod; every other day.

Statistical analyses were conducted using GraphPad Prism9 (GraphPad). A 2-tailed unpaired Student’s *t* test was performed when comparing 2 sets of data (Figs. 1e, 2d, 2e, 3c, 3e, 4d, 4f, 5b). One-way ANOVA followed by Tukey’s multiple comparison test was performed when comparing more than 2 sets of data (Fig.s 4a, 5e, 5f, 6c, 6f, 6g). A Brown-Forsythe and Welch ANOVA test followed by Dunnett’s T3 multiple comparison test was performed for Fig. 1b. Linear regression analyses were performed for Figs. 3c and 4d. *P* < .05 was considered statistically significant.

**Figure 6.**
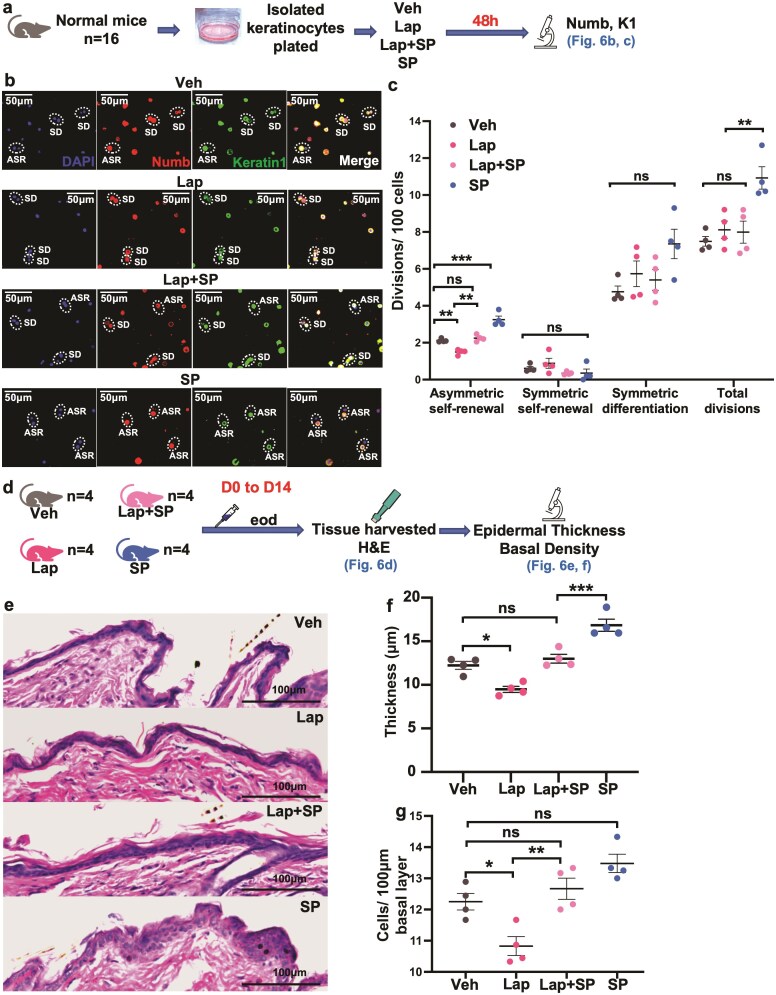
Asymmetric SC divisions are required for substance P-induced stratification. (**a**) Treatment of normal keratinocytes with lapatinib, substance P, or lapatinib and substance P, in vitro. (**b**) Effect of lapatinib on substance P-induced asymmetric SC self-renewal in vitro (*n* = 4). Division fate analysis using Numb and keratin 1 immunofluorescence. Representative images. (**c**) Effect of lapatinib on substance P-induced asymmetric SC self-renewal in vitro. (**d**) Experimental design: treatment of normal mice with lapatinib, substance P, or lapatinib and substance P, in vivo. (**e**) Histology showing the effect of lapatinib on substance P-induced epidermal thickening. (**f**) Thickness (µm) of murine skin treated with lapatinib, substance P, or lapatinib and substance P (*n* = 4). (**g**) Effect of substance P and lapatinib on basal cell density (cells per 100 µm basal layer). Data are presented as mean ± SEM; *P < 0.05. **P < 0.01. ***P < 0.001. ns; not significant. Veh; vehicle. Lap; lapatinib. SP; substance P. eod; every other day.

## Results

### Both Perpendicular (Asymmetric SC Self-Renewal) and Parallel Basal Layer Divisions, But Not Suprabasal Divisions, Are Increased at Day 4 Post-wounding

To assess divisions in the basal layer during wound healing, wounds were made on the flanks of mice. Wounds healed in 9.3 ± 0.5 days (Fig. 1a). Four days after wounding, during the period of maximal proliferation,^14^ tubulin expression was used to study cell divisions (Fig. 1b-1e). A total of 89.4 cm of intact skin (mean 22.3 cm, *n* = 4) and a total of 76.5 cm of wounded skin (mean 19.1 cm, n = 4) were examined. Hyperproliferation was seen in the region from 0 to 4 mm from the leading edge (Fig. 1b, center). Basal layer divisions were increased. While suprabasal divisions are uncommon in adult murine epidermis, we examined for evidence of increased suprabasal divisions in response to wounding. No significant increase was detected in dividing cells appearing to have no connection with the basal membrane, and thus we did not perform further studies to confirm their suprabasal nature. In the basal layer, perpendicular divisions were increased 5-fold and parallel divisions 2-fold (Fig. 1e). Thus, the early response to wounding is an increase in basal layer divisions, not suprabasal divisions.

### The Hyperproliferation After Wounding Is Associated With an Increase in Both Asymmetric and Symmetric SC Self-Renewal, With No Detected Change in CP Divisions

In vitro analysis allows symmetric self-renewal of SCs to be distinguished from symmetric differentiation of CPs; keratin 1 and Numb are absent in both daughters of symmetric SC self-renewal divisions, and present in both differentiated daughters of symmetric differentiation (Fig. 2a, 2b).^31,40,41^

At day 4 post-wounding, skin was harvested in a 4mm radius around the wound to include the proliferative zone (Fig. 1b). Control skin was harvested from littermates. Keratinocytes were isolated, plated for 48 h, and cell divisions assessed (Fig. 2c). Wounding increased both asymmetric SC self-renewal and symmetric SC self-renewal. No significant change in symmetric differentiation divisions (CP) was detected (Fig. 2d). Also, no significant change was detected in the proportions of asymmetric and symmetric self-renewal before and after wounding (Fig. 2e). Thus, our findings in vivo were matched by the in vitro findings (Fig. 1e cf. Fig. 2d); the early response to wounding is in the SC, not the CP, and the balance of asymmetric/symmetric SC self-renewal is not found to change.

### In Mice With Decreased Sensory Innervation, the Decreased Proliferative Response to Wounding Is Associated With a Decrease in Asymmetric SC Self-Renewal and CP Symmetric Differentiation

We confirmed delayed wound healing with decreased sensory innervation in our model (Fig. 3a-3c). Neonatal mice were treated with capsaicin (Fig. 3a) and the tail-flick method used to confirm loss of sensation, typically occurring by day 18 (Supplementary Table S1). In mice with decreased sensory innervation, wound healing was not complete until 24.8 ± 0.3 days versus vehicle-treated mice 14.5 ± 0.3 days (*P* < 0.0001). The rate of healing appeared most dramatically inhibited from days 0 to 3, but the rate of healing overall was significantly decreased (slopes −1.1*x* ± 0.02 vs. −1.9*x* ± 0.07, *P* < 0.0001; Fig. 3c). It may be noted that the rate of healing for the vehicle-treated mice (Fig. 3b) is considerably slower than for the control in Fig. 1a. Tween in the vehicle (Tween:ethanol:saline 1:1:8vol) is an anionic surfactant, likely responsible for the delay in healing time seen. Anionic surfactants are accepted as potent irritants to human and animal skin.

Cell division fate was analyzed post-wounding (Fig. 3a, 3d, 3e). In vitro, decreased sensory innervation was associated with decreased asymmetric SC self-renewal and symmetric differentiation (Fig. 3d, 3e). Decreased asymmetric SC self-renewal would be expected to result in a decrease in CP divisions (symmetric differentiation) over time. Thus, whether the decrease in CP divisions is a direct effect of loss of innervation on CPs, or secondary to the decreased asymmetric SC self-renewal (over the denervation period), cannot be determined here.

### Substance P Improves Wound Healing in Mice With Decreased Sensory Innervation and Increases Keratinocyte Asymmetric SC Divisions and CP Symmetric Differentiation Divisions In Vitro

Decreased sensory innervation results in depletion of substance P^22-24^ and substance P improves wound healing in skin.^5,26-29^ We therefore studied whether substance P would reverse the decreased asymmetric SC self-renewal in seen in response to wounding in skin with decreased sensory innervation. Various doses of substance P have been used.^37,42,43^ Our study showed that keratinocyte proliferation was maximally stimulated at 10^−7^ M (Fig. 4a).

To study substance P and wound healing in skin with decreased sensory innervation, mice were treated with substance P or vehicle, injected intradermally around the wound^43^ every other day, for 10 days before wounding and during wound healing (Fig. 4b). In skin with decreased sensory innervation, vehicle-treated wounds completely healed in 24.0 ± 0.3 days, while substance P-treated wounds completely healed in 18.8 ± 0.3 days (*P* < 0.0001; Fig. 4c, Supplementary Fig. S2). At all timepoints, substance P-treated wounds showed a lesser area than vehicle-treated wounds (Fig. 4d) and the rate of healing was increased with substance P vs. vehicle treatment (slopes −1.75 ± 0.17 vs. −1.27 ± 0.04, *P = *0.01). Tween, an anionic surfactant in the vehicle, was likely responsible for the delay in healing time in the vehicle (Fig. 4d, 4e) vs. untreated controls in Fig. 3b, 3c. Anionic surfactants are broadly accepted as potent irritants to human and animal skin.

Division fate analysis showed that substance P treatment of wounds with decreased sensory innervation was associated with increased asymmetric self-renewal and symmetric differentiation, while no change in symmetric self-renewal was detected (Fig. 4e, 4f). However, the increase in CP symmetric differentiation could be either a direct or indirect effect of substance P on CPs, because the studies followed 10 days of substance P treatment in vivo before wounding. Nonetheless, these studies demonstrate that Substance P increases asymmetric SC self-renewal in normal keratinocytes and effectively reverses decreases in asymmetric SC self-renewal and symmetric differentiation caused by decreased sensory innervation. Therefore, substance P secretion by sensory neurons contributes to asymmetric SC self-renewal and differentiation during wound healing in normal skin.

### Substance P Administration Can Ameliorate the Decreases in Perpendicular and Parallel Basal Layer Divisions in Non-wounded Skin With Decreased Sensory Innervation

We then assessed cell divisions in non-wounded skin with and without decreased sensory innervation. For intact skin a total of 52.8 cm (mean 17.6 cm, *n* = 3) was examined and for skin with decreased sensory innervation a total of 125.7 cm (mean 41.9 cm, *n* = 3) was examined.

In non-wounded skin with decreased sensory innervation, both perpendicular and parallel divisions in the basal layer of the intact skin were decreased and no suprabasal divisions were seen (Fig. 5a, 5b); not surprising given that in most areas the epidermis consisted of a single layer.

It is believed that asymmetric SC self-renewal is key for stratification of the epidermis.^9-12^ We therefore treated mice with decreased sensory innervation and control mice with Substance P or vehicle every other day for 24 days and then examined the epidermis (Fig. 5c). We found that substance P-induced asymmetric SC self-renewal was associated with increased epidermal stratification in both normal skin and skin with decreased sensory innervation (Fig. 5d, 5e). In normal skin, substance P-induced asymmetric self-renewal was associated with a thickened epidermis and no detected change in basal cell density (Fig. 5f). However, in skin with decreased sensory innervation (decrease in both perpendicular and parallel divisions of the basal layer, a thin epidermis, and reduced basal cell density), substance P restored stratification and basal cell density. This may have occurred by either increased CP divisions in the basal layer or by the daughters of asymmetric divisions ending up in the basal layer, as previously described in developing epidermis.^44^

### Asymmetric SC Divisions Are Required for Substance P-Induced Stratification

To test whether asymmetric SC divisions are required for substance P-induced increased epidermal stratification, we used Laptatinib to inhibit asymmetric SC self-renewal in vivo, and examined epidermal stratification.Lapatinib, an EGFR (epidermal growth factor receptor) inhibitor, was shown to reduce asymmetric SC divisions in muscle by 65%.^39^ Similarly, administration of lapatinib to substance P-treated or normal keratinocytes in vitro were both associated with a decrease in asymmetric SC self-renewal (Fig. 6a-6c). In vivo, treatment with lapatinib in combination with substance P over 14 days prevented the increase in epidermal stratification induced by substance P and resulted in thinning of normal vehicle-treated epidermis (Fig. 6d-6f). Thus, lapatinib reduced substance P-induced asymmetric self-renewal and prevented increased stratification.

During development, it has been noted that following perpendicular divisions (asymmetric SC self-renewals) the suprabasal daughter cell may end up in the basal layer.^44^ In skin with decreased sensory innervation, substance P restored basal cell density to normal (Fig. 5f), suggesting that this may occur post-development also. However, the increase in asymmetric SC self-renewal related to substance P in normally sensate adult skin (normal basal cell density) did not produce a detected significant change in the basal cell density in this study (Fig. 6g), suggesting that the perpendicular divisions were exclusively stratifying the epidermis when a normal density of basal cells was present.

## Discussion

These studies were designed to define the contributions of SC and CP proliferation to wound healing and to elucidate the effects of substance P on SC and CP behavior. Unexpectedly, results of the studies with substance P and decreased sensory innervation also showed a role for asymmetric divisions in epidermal stratification in vivo.

Our examination of divisions peripheral to the wound’s leading edge showed that early proliferation after wounding occurs in the basal, not suprabasal, layer in keeping with findings in mouse tail.^14^ In vitro studies, that allowed us to distinguish symmetric SC self-renewal from symmetric differentiation (predominantly CP divisions)^31^ similarly did not show a significant increase in symmetric differentiation. These findings indicate that early post-wounding hyperproliferation is happening primarily in SCs not CPs. This contrasts with studies in cornea, where early after wounding there was increased CP proliferation, with CPs undergoing their full replicative potential and a shortening of cell cycle duration.^13^

SCs self-renew to produce new copies of themselves via symmetric division (2 copies) or asymmetric division (1 copy), or differentiate into 2 CPs via symmetric differentiation. A tight balance of these division fates is critical for the functional integrity of the epidermis. In human psoriasis and murine psoriasis models, increased asymmetric SC self-renewal divisions are associated with increased CPs and no change in SC number.^8^ Likewise, increased asymmetric SC divisions underly murine inflammatory bowel disease.^45^ In contrast, squamous cell carcinoma is associated with predominantly symmetric divisions^8^ and in multiple aggressive tumors symmetric SC self-renewal is a driving oncogenic feature.^46,47^ Also, underlying hypoproliferative aging epidermis^31^ and blood^48^ there is a decrease in asymmetric SC divisions.

We hypothesized that the response to wounding, like the expanding skin in development, would involve increased symmetric SC self-renewal. This seemed in keeping with squamous cell carcinomas occurring in long-standing wounds. However, analysis of keratin 14-derived clones at the edge of wounds suggested that SCs undergo asymmetric division^14^ in murine tail skin. Our studies here indicate a proportionally equivalent increase in asymmetric and symmetric SC self-renewal in re-epithelialization in adult murine truncal epidermis.

Skin with decreased sensory innervation displayed an attenuated proliferative response to wounding and substance P was highly effective at improving re-epithelialization. Substance P increased both asymmetric SC self-renewal and symmetric differentiation, indicating that innervation is important for SC function and substance P appears to have a major role. While further work will be needed to determine how substance P promotes asymmetric SC divisions, substance P acts through G-Protein coupled neurokinin receptors and both substance P and its receptor NK1R are expressed in different immune cell types^49^ and in keratinocytes.^50^ Thus, the action of substance P may be direct activation of NK receptors in keratinocytes and/or indirect, as part of substance P promoted inflammation and cytokine secretion.^51,52^

Substance P, along with CGRP, VIP, neuropeptide Y, and neurotensin all affect wound healing, and substance P, CGRP, and VIP stimulate epidermal organization and keratinocyte proliferation.^53^ Neuropeptides modulate the cytokine response (IL-1, IL-6, IL-8, IL-10, and TNF-α) to wound healing.^54,55^ Substance P has emerged as a potent modulator of cutaneous wound healing among associated neuropeptides.^54^ Here, our studies clearly show the capacity of substance P to rescue SC defects in wound healing that result from reduced cutaneous innervation, suggesting that amongst the broader family of neuropeptides, substance P is a biologically-relevant contributor to SC activation during wound healing.

An evolving concept has been that asymmetric SC divisions are responsible for epidermal stratification, and there is much support for this concept. However, recent studies in developing^44,56,57^ and adult^44,56,58,59^ murine skin bring into question the coupling of stratification and division orientation of basal progenitors.

In development, homeostasis, and disease, changes in the balance of asymmetric and symmetric SC self-renewal divisions occur. During murine development, symmetric divisions predominate until stratification begins, at which time asymmetric SC self-renewal predominates.^10,60^

In human psoriasis and murine psoriasis models, increased asymmetric SC self-renewal divisions are associated with a profound increase in stratification.^8^ Aging human epidermis undergoes a decrease in asymmetric SC divisions and epidermal stratification.^31^ Further support for this association comes from experiments deleting key polarity proteins. Loss of LGN or PDK1 in murine epidermis reduced asymmetric SC divisions and resulted in a thinner epidermis.^12,61^ Loss of aPKCλ increased asymmetric SC divisions and epidermal stratification increased.^62^ The above findings support the contention that asymmetric SC self-renewal results in epidermal stratification in development and disease.

However, it is also proposed that mechanisms other than SC division fate are responsible for stratification of the epidermis under various circumstances. In developing skin, the correlation between stratification and division orientation can be explained by the topology of the epidermis restraining division orientation, rather than the opposite causal relationship,^44^ ie, asymmetric SC self-renewal is only permitted by a multilayered epidermis. Studies of murine embryonic development suggest that cell crowding within the basal layer is a factor in increasing asymmetric divisions.^56^ More relevant to adult skin perhaps, in homeostatic adult epidermis differentiation of neighboring cells drives SC self-renewal.^58^

We tested whether asymmetric SC self-renewal is required for the increased stratification seen with substance P administration. Lapatinib inhibited asymmetric self-renewal in favor of symmetric SC self-renewal in muscle^39^ and in our studies. Lapatinib prevented substance P-induced asymmetric divisions and the associated increased epidermal stratification, indicating that increased asymmetric divisions are required for the change in skin stratification in normal adult skin. The in vitro experiments showing increased asymmetric SC self-renewal with substance P administration, both in mice with decreased sensory innervation and in intact mice (Figs. 4e, 4f and 6b, 6c, respectively) are free from the influences of crowding and other external cues of the basement membrane and the niche, but still demonstrate that substance P increases asymmetric SC divisions and CP divisions. Interestingly, the results of our in vitro studies closely match results of the ex vivo studies. While in vitro studies may reflect intrinsic cues, our findings do not eliminate the possibility that a replete basal layer is a factor in the increased perpendicular divisions observed,^56,57^ as extrinsic as well as cell intrinsic factors influence division type.

Likewise, a decrease in basal cell density could contribute the observed decrease in asymmetric self-renewal divisions ex vivo due to either lapatinib or capsaicin.^57^ However, again, in vitro where cells did not experience crowding, lapatinib led to a decrease in asymmetric divisions.

In developing skin, the differentiated cell of asymmetric self-renewal divisions may end up in the basal layer^44^ and divisions in the basal layer were found to occur in response to neighbor cell delamination.^63^ Substance P treatment of epidermis with decreased sensory innervation returned both basal cell density as well as epidermal stratification to normal, suggesting that, likewise, deficient basal cell density in adults might be ameliorated by both daughters of asymmetric SC self-renewal divisions ending up in the basal layer. Recent work modeling tissue stress and deformation shows how biomechanical signaling can play a role in delamination and maintaining basal cell density.^64^ Thus, many recent studies are elucidating factors that may work in synchrony to determine basal cell density, delamination, and stratification under various circumstances.

In previous work, perpendicularly oriented divisions in tissue sections were found to be asymmetric^10,12^ and our in vitro studies reflect similar numbers of asymmetric divisions in vitro as perpendicular divisions in vivo. However, in studies of organogenesis^65^ post metaphase reorientation of spindles occurred, and a limitation of our studies is that this cannot be excluded in the ex vivo studies presented here.

In summary, these studies provide evidence that the proliferative response early in wound healing is in the SC rather than CP compartment, and that the response is a proportionally equivalent increase in asymmetric and symmetric SC self-renewal. Decreased sensory innervation is associated with a decrease in both SC and CP proliferation. Substance P induced proliferation through an increase in asymmetric SC divisions and in CP divisions, restored basal cell density, and increased stratification of the epidermis. Inhibition of asymmetric SC self-renewal (lapatinib) prevented substance P-induced stratification, suggesting that increased asymmetric SC divisions are required for the increase in epidermal stratification.

## Supplementary Material

Supplementary material is available at *Stem Cells* online.

sxae009_Supplementary_Data

## Data Availability

Datasets related to this article can be found at https://doi.org/10.6084/m9.figshare.22280254.v1, an open-source online data repository hosted at Figshare (Khalifa et al, 2023).
