# Supplementary material for: Neuropeptide substance P alters stem cell fate to aid wound healing and promote epidermal stratification through asymmetric stem cell divisions
Source: Stem Cells. 2024 Feb 1;44(3):sxae009. doi: 10.1093/stmcls/sxae009 (PMC13017499; doi:10.1093/stmcls/sxae009)
Supplement: sxae009_Supplementary_Data [file sxae009_supplementary_data.pdf]

| Time of response to heat stimulus (seconds) | | | | | | | | |
|---|---|---|---|---|---|---|---|---|
| | Vehicle | | | | Capsaicin | | | |
| | Mouse 1 | Mouse 2 | Mouse 3 | Mouse 4 | Mouse 1 | Mouse 2 | Mouse 3 | Mouse 4 |
| Day 0 | 2 | 3 | 3 | 3 | 2 | 3 | 3 | 3 |
| Day 3 | 3 | 3 | 3 | 4 | 3 | 3 | 4 | 4 |
| Day 6 | 4 | 2 | 3 | 4 | 5 | 4 | 5 | 4 |
| Day 9 | 3 | 2 | 2 | 4 | 7 | 5 | 7 | 5 |
| Day 12 | 3 | 3 | 2 | 3 | 9 | 7 | 7 | 7 |
| Day 15 | 3 | 3 | 2 | 4 | 9 | 9 | 8 | 8 |
| Day 18 | 3 | 4 | 3 | 4 | 10 | 10 | 9 | 10 |
| Day 21 | 3 | 4 | 3 | 4 | 10 | 11 | 9 | 10 |

**Supplementary Table 1: Tail flick test to confirm decreased sensory innervation.** The tails of capsaicin-treated mice were placed in a water bath maintained at 52℃. The latency time until tail withdrawal was recorded. A time of 10 seconds or greater was considered evidence of decreased sensory innervation.

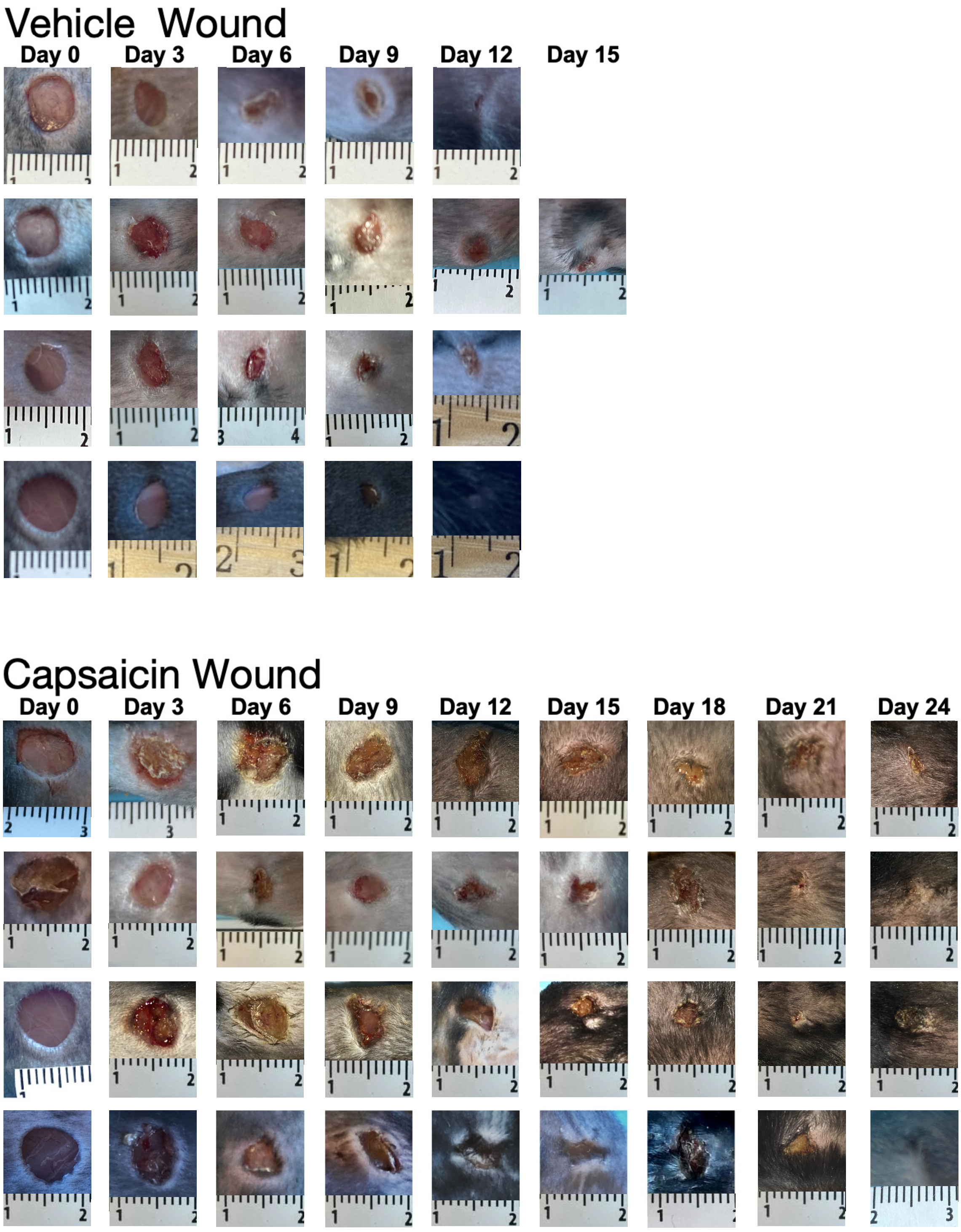


**Supplementary Figure 1: Effect of decreased sensory innervation on wound healing in mice.** Wounds were photographically monitored every 3 days till complete healing.

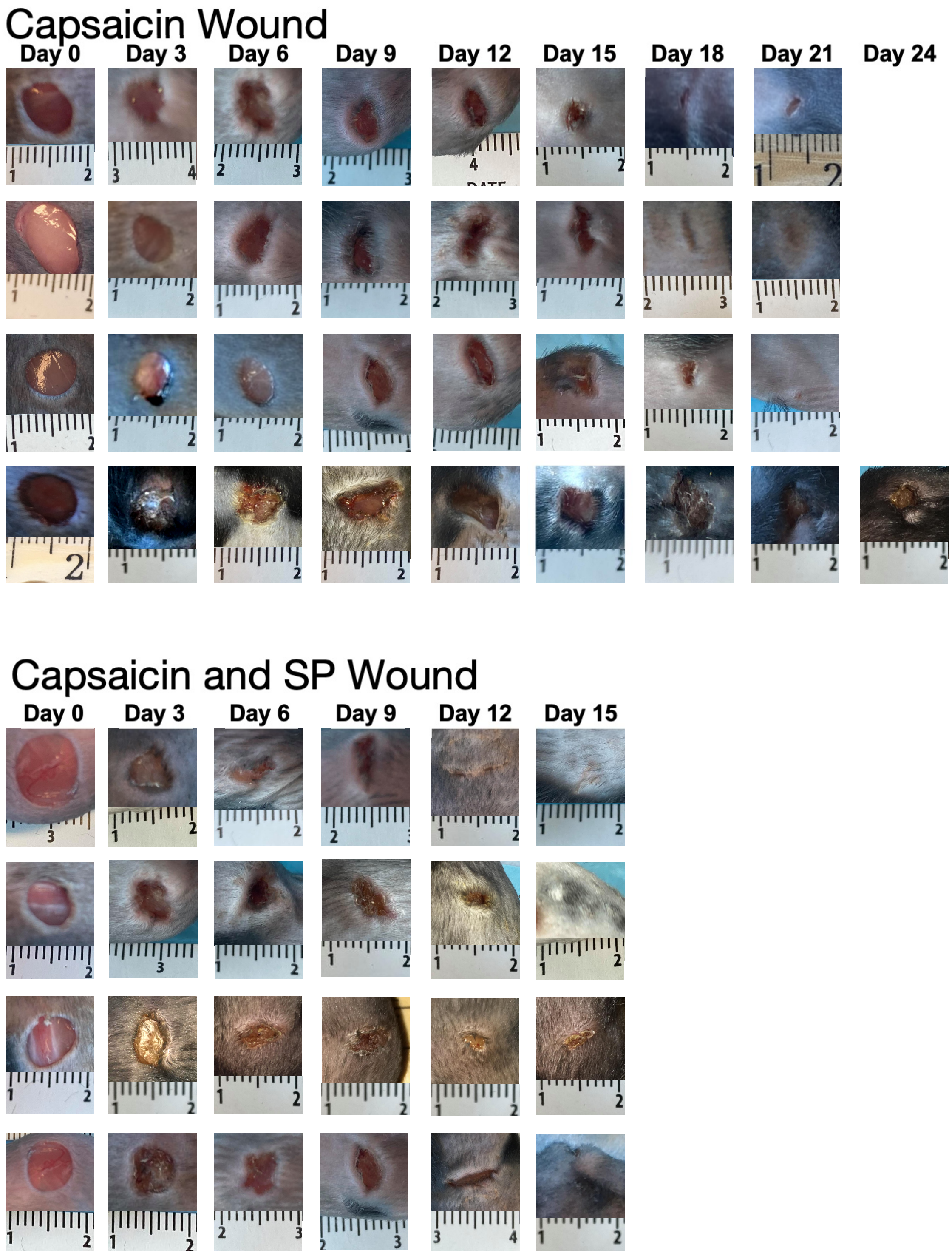


**Supplementary Figure 2: Effect of substance P on wound healing in mice with decreased sensory innervation.** Wounds were photographically monitored every 3 days till complete healing.
